# The Local Representative

**Published:** 1918-09-21

**Authors:** 


### THE MATRONS' AND SISTERS' DEPARTMENT.

### THE LOCAL REPRESENTATIVE.

The hinge upon which every local centre of the College of Nursing turns is its local representative. This honorary officer is the necessary link between the College and its offshoots. She has definite functions with regard to the College and her own centre. She is often the moving spirit in founding the centre, and she is responsible for keeping it in close touch with the parent College. Unless another chairman be specially appointed, as at Liverpool and Bristol, the local representative is chairman at all the meetings of the local centre, and if unable to be present is charged with the duty of providing a substitute. It is she who directs the calling of meetings of committees (other than of the Finance Committee) and of members. It is to her that members are directed to apply in difficulties. She is thus an indispensable part of the local development of the centre. While acting in entire conformity with the decisions of the local Executive Committee over which she presides, she is the authority to whom it is natural to turn in matters affecting the centre.

But the functions of the local representative are by no means exclusively confined to the affairs of the local centre. It is one of the main problems in all decentralisation schemes, such as this movement for forming strong provincial nursing centres, to ensure that the current of vitality flows freely between the main trunk and its offshoots. The method by which this mutual exchange is brought about in the College of Nursing is the Standing Committee of Local Representatives, presided over by a member of the College Council, meeting at stated intervals and when circumstances require. This Standing Committee is a very important link not merely between each local centre and the College, but between local centres all over the kingdom. Its future is all to make. So far its discussions have been but tentative, and since the number of local representatives has been rapidly added to during the past year it can hardly yet be said to have found itself. Yet even in its beginnings it is proving fruitful in suggestion and criticism, in the examination of local difficulties and the best methods of meeting them, as well as in removing the sense of isolated endeavour which is apt to seize on representatives of newly formed centres. It may be interesting to our readers to have a list of the centres hitherto formed and now in working order, with the names of their local representatives on the Standing Committee. We give them in alphabetical order.

The chairman of the Standing Committee is Miss Sparshott. The local representatives are as follows:—Birmingham, Miss A. M. Bodley (hon. secretary); Bristol, Miss McHardy; Miss A. Baillie (chairman); Derby, Miss Sutcliffe; Leeds, Miss Innes; Leicester, Miss Masters; Liverpool, Miss C. Worsley, Miss Cummins (chairman); Manchester, Miss Sparshott; Plymouth, Miss Dickson; Sheffield, Miss Smeeton; Truro, Miss Chaff. The centres in actual working order at the end of the summer were ten in number. To these must be added Newcastle and Cambridge, founded in September. Several others will shortly, it is hoped, be added.

It is probable that the duties performed by local representatives in their own centre will vary in each locality. Without exception they are all busy people. As the centres become larger the work will increase and a sub-division of labour will be called for. These matters of procedure are being tackled with patience and common sense, by women well accustomed to affairs. The assignment of various branches of work to the hon. secretaries and treasurer is capable of considerable variation in practice. By a very sensible arrangement the local representative is enabled to keep in touch with her Standing Committee even should she be prevented from attendance at it. She is permitted to appoint a deputy, approved by her committee, to attend in her place, and by this means the Standing Committee is sure of a good attendance and the local centre is not deprived by the accident of unforeseen circumstances from participation in debates affecting its interests. It happens too often that members of committees meeting at a distance find themselves discouraged by frequent inability to attend, and so lose all interest in the proceedings. The appointment of alternative members with power to vote is therefore a valuable safeguard.

